# Incidence of hepatopathies in dogs administered zonisamide orally: A retrospective study of 384 cases

**DOI:** 10.1111/jvim.16398

**Published:** 2022-03-02

**Authors:** Tess K. Smith, Starr Cameron, Lauren A. Trepanier

**Affiliations:** ^1^ University of Wisconsin ‐ Madison Madison Wisconsin USA

**Keywords:** antiepileptic drug, epilepsy, seizures, adverse effects

## Abstract

**Background:**

Acute hepatopathy secondary to administration of zonisamide has been reported in 2 dogs, but overall incidence of hepatopathy is unknown.

**Objective:**

To characterize the incidence of hepatopathy in dogs administered zonisamide PO.

**Animals:**

Three hundred eighty‐four dogs administered zonisamide PO.

**Methods:**

Multicenter retrospective study. Medical records were searched for dogs prescribed zonisamide PO and which had follow‐up for at least 3 months (acute exposure) and >3 months (chronic exposure). Reported clinical signs, physical examination findings, and serum biochemical panels were reviewed for possible hepatotoxicosis. Serum alanine aminotransferase (ALT) and alkaline phosphatase (ALP) activity and albumin concentration were documented for all available cases.

**Results:**

Acute clinical hepatopathy was found in 2 of 384 treated dogs (0.52%, 95% confidence interval [CI], 0.06‐1.9) after 13‐16 days of zonisamide treatment. One additional dog had elevated serum ALT activity with no clinical signs. Of these 3 dogs, 2 recovered after administration of zonisamide was stopped, and 1 was euthanized because of liver failure. Of the 117 cases chronically administered zonisamide, 10 had an increase in ALP, 6 had an increase in ALT, and 1 had hypoalbuminemia. No clinical signs of liver disease were noted in dogs chronically treated with zonisamide (median, 20 months; range, 5‐94 months).

**Conclusions and Clinical Importance:**

Acute, potentially life‐threatening hepatopathy associated with oral administration of zonisamide to dogs is estimated to occur in less than 1% of dogs and was observed in the first 3 weeks of treatment. Subclinical abnormalities in ALT and ALP activity were noted in <10% of dogs during chronic administration of zonisamide, with no clinical signs of liver disease noted.

**Abbreviations**
AED: antiepileptic drugs
ALP: alkaline phosphatase
ALT: alanine aminotransferase

## 1 INTRODUCTION

Zonisamide is considered a newer antiepileptic drug (AED) and is becoming commonly used for treating seizures in dogs. Zonisamide is currently labeled for adjunct treatment of partial seizures in humans.^1^ In dogs with epilepsy, zonisamide is used for both monotherapy and adjunct management of seizures.^1^ Limited studies in dogs have reported 60% efficacy when used as monotherapy and 58% to 80% as adjunct treatment.^2^, ^3^, ^4^

Zonisamide is generally well‐tolerated in dogs, and the most common adverse effects include decreased appetite, vomiting, sedation, and ataxia.^1^ Idiosyncratic drug reactions occur, including neutropenia, erythema multiforme, and acute hepatopathy.^1^ Specifically, 2 cases of severe, acute, idiosyncratic hepatopathy are reported in dogs.^5^, ^6^ In addition, persistent increases in serum alkaline phosphatase (ALP) activities and decreases in serum albumin concentration can occur in dogs treated experimentally with zonisamide at 5 times the recommended clinical dose (dosages used were 75 mg/kg/day).^7^ However, the incidence of hepatopathies has not been evaluated in dogs treated clinically acutely or chronically with zonisamide.

The purpose of this retrospective study was to characterize the incidence of a hepatopathy associated with acute and chronic zonisamide administration in dogs with a history of seizures.

## 2 MATERIALS AND METHODS

### 2.1 Study cohort

The study design was a multicenter retrospective study. The medical records for dogs presented to Sage Veterinary Centers (Redwood City, Campbell, Dublin, and Concord, CA) and administered zonisamide PO from 2011 to 2018 were reviewed.

### 2.2 Hepatopathy associated with acute zonisamide administration

For evaluation of the incidence of hepatopathy associated with acute zonisamide administration in dogs, inclusion criteria included dogs of any age, any epilepsy diagnosis, and at least 3 months of clinical follow‐up after starting zonisamide. Dogs could be administered other medications at the time of first administration of zonisamide, but baseline bloodwork was required before oral administration of zonisamide, and dosing of other drugs had to remain unchanged during the 3‐month observation period. Adverse clinical signs noted during the 3‐month period were abstracted from the medical record, including anorexia, hyporexia, vomiting, diarrhea, excessive lethargy, or icterus. Dogs were excluded if they had been diagnosed hepatopathy or concurrent systemic illness that could confound these clinical signs. Serum biochemical panels, if performed during this 3‐month period, were reviewed for any abnormalities in alanine transferase (ALT) or ALP activity or albumin concentration. In order to maintain an accurate “denominator” for the number of treated dogs, performance of bloodwork was not required if clinical records indicated a clinically well dog at that visit.

### 2.3 Hepatopathy associated with chronic administration of zonisamide

To evaluate for evidence of hepatopathies associated with long‐term zonisamide administration, dogs receiving zonisamide PO for longer than 3 months were also included. For inclusion, follow‐up blood work at any time point after first administration of zonisamide, including ALT and ALP activity and albumin concentration, was required. If any dog had an increased ALT or ALP activity, or decreased albumin concentration, documentation of normal values before starting zonisamide was required. Dogs concurrently receiving prednisone or phenobarbital were excluded, because of the induction of liver enzyme activity by these drugs.^8^, ^9^, ^10^, ^11^ Additionally, any dog diagnosed with a pre‐existing hepatopathy or other systemic illness was excluded.

### 2.4 Statistical analysis

Descriptive data are reported as percentages with 95% confidence intervals (CIs) for acutely treated dogs. The incidence of possible hepatopathy was expressed as a percentage by dividing the total number of cases with clinical or biochemical signs of acute hepatopathy by the total number of treated dogs that fit the inclusion criteria. For chronically treated dogs, the incidence of increases in ALT activity, increases in ALP activity, or decreases in albumin concentration was also calculated as percentages with 95% CI, using the total number of treated dogs with any biochemical screening as the denominator.

## 3 RESULTS

### 3.1 Study cohort

After searching the electronic medical records system for dogs prescribed zonisamide at our hospital sites, 788 cases were identified and reviewed, and 384 dogs met the inclusion criteria for acutely treated dogs. The mean age was 6.2 years (range, 6 months to 17 years, SD 4.1) and mean weight was 19.1 kg (range, 1.6‐63.6 kg, SD 13.1). For chronically treated dogs, 117 dogs met the inclusion criteria. The mean age was 4.6 years (range, 8 months to 16 years, SD 3.2) and mean weight was 18.4 kg (range, 2.3‐74.2 kg, SD 13.9).

### 3.2 Apparent incidence of clinical or biochemical hepatopathy associated with acute administration of zonisamide

Two, or 0.52%, of 384 dogs (95% CI, 0.06‐1.9) that were clinically monitored developed clinical signs along with biochemical evidence of acute hepatopathy during the first 3 months of treatment with zonisamide. Of the 103 dogs with biochemical monitoring, 1 dog developed changes consistent with an acute hepatopathy without any clinical signs noted, for an overall incidence of biochemical hepatopathy of 2.9% (3 of 103 dogs).

Dog 1 was a 15‐year‐old female spayed Australian Shepherd that presented for a 1‐month history of seizures and had been diagnosed with an extra‐axial olfactory mass. Baseline blood work showed an ALT of 164 U/L (reference range, 10‐125 U/L) and an ALP of 911 U/L (reference range, 23‐212 U/L). The dog was administered zonisamide (6.3 mg/kg q12 PO). Abdominal ultrasound performed 9 days later noted the liver was slightly decreased in size and no other abnormalities were detected. The dog administered prednisone (0.57 mg/kg/day PO) for the olfactory mass. At 16 days, the owners reported a decreased appetite and lethargy. At this time, ALT was 756 U/L and ALP was 1060 U/L. She was administered S‐adenosylmethionine (19.3 mg/kg q24 PO). On day 23, she presented for a continued decreased appetite and 2 episodes of vomiting. ALT was 3162 U/L, ALP was 10 679 U/L, albumin was 2.8 g/dL (reference range, 2.7‐3.9 g/dL), and total bilirubin was 0.6 mg/dL (reference range, 0.0‐0.3 mg/dL). Zonisamide dosage was tapered over 7 days and prednisone was continued. ALT was 2115 U/L and ALP was 10 222 U/L. Liver aspirates revealed mild to moderate vacuolar change and mixed lymphocytic inflammation. Clinical improvement was noted 11 days after complete discontinuation of zonisamide, despite continuation of prednisone at the original dose. At 30 days, the dog was clinically normal. Her ALT was 179 U/L and ALP was 971 U/L and the prednisone dose remained the same during this treatment and monitoring period.

Dog 2 was an 8‐year‐old female spayed Miniature Schnauzer that presented for acute onset cluster seizures. Baseline ALT was 224 U/L (reference range, 12‐118 U/L) and ALP was 144 U/L (reference range, 5‐131 U/L), and she was started on zonisamide (7.8 mg/kg q12 PO) and levetiracetam (39 mg/kg q8 PO). Thirteen days after starting zonisamide, the dog presented for acute onset vomiting and anorexia. Levetiracetam had been discontinued 2 days before presentation. Blood work revealed an ALT of 2170 U/L, ALP of 1916 U/L, and total bilirubin of 11.8 mg/dL (reference range, 0.0‐0.3 mg/dL). Serum albumin concentration was 3.1 g/dL (reference range, 2.7‐3.9 g/dL). Prothrombin time and partial thromboplastin time were both prolonged (78 and 173 seconds, respectively). On abdominal ultrasound, the liver was of normal echogenicity and size. She was treated with fluid therapy, antiemetics, ampicillin (22 mg/kg IV q8), metronidazole (7.5 mg/kg IV q8), and N‐acetylcysteine (140 mg/kg IV once). Her mentation and clinical response continued to decline over the 12‐hour hospitalization, and humane euthanasia was elected. No necropsy was performed.

The third dog, a 4‐year‐old male neutered French Bulldog, developed biochemical evidence of hepatopathy 15 days after administering zonisamide (7.1 mg/kg q12 PO) without clinical signs. Before administering zonisamide, ALT activity was 186 U/L (reference range, 10‐125 U/L) and ALP activity was 56 U/L (reference range, 23‐212 U/L). Fifteen days after zonisamide administration, ALT activity was 896 U/L, ALP activity was 117 U/L, and albumin concentration was 3.5 g/dL. Levetiracetam was added as an AED, and zonisamide treatment was discontinued. Repeat measured serum ALT activity was within the reference range (96 U/L) 2 weeks after discontinuing zonisamide.

### 3.3 Incidence of possible biochemical hepatopathy associated with chronic administration of zonisamide

One hundred seventeen dogs met the inclusion criteria. The median follow‐up time after administering zonisamide was 20 months and 18 days (range, 5‐94 months). No dogs developed clinical signs consistent with liver disease. Ten out of 117 dogs (8.6%; 95% CI, 4.2‐15.2) developed a further increase in ALP alone compared to their baseline values (range, 40‐126 U/L before zonisamide, 134‐746 U/L after zonisamide). None of these dogs had follow‐up diagnostics performed. Six out of 117 dogs (5.1%; 95% CI, 1.9‐10.8) had a new but mild increase in ALT alone compared to their baseline (range, 27‐73 U/L before zonisamide, 125‐200 U/L after zonisamide). In 1 of these dogs, repeat measurement revealed that ALT had normalized within 8 months with no changes in treatment. One dog had an increase in both ALT and ALP activity (126 and 476, respectively) with no clinical signs and no further diagnostics performed. Only 1 out of 117 dogs (0.85%; 95% CI, 0.02‐4.7) had a low serum albumin concentration alone (2.7 g/dL before zonisamide, 2.5 g/dL after zonisamide), with 1+ protein on a urinalysis. No dogs developed a low albumin concentration in combination with increases in ALT or ALP activity, and no dogs developed hyperbilirubinemia with chronic dosing of zonisamide.

## 4 DISCUSSION

This study investigates the incidence of hepatopathies administered zonisamide PO in pet dogs. An acute, clinical hepatopathy is reported in 2 isolated case reports in the veterinary literature. We found the incidence of acute, clinical hepatopathy to be rare, with an incidence of 0.57% in dogs receiving zonisamide administered PO. The detected overall incidence of acute biochemical hepatopathy was 2.9%; however, this is an estimate only because not all dogs had consistent biochemical monitoring. Although none of the 3 affected dogs underwent liver biopsies, the chronology of the increases in liver enzyme activity, along with resolution after discontinuation of zonisamide in 2 surviving dogs, are consistent with an acute adverse drug reaction to zonisamide. Given the low incidence and the time to onset in our study (13‐16 days) and in 2 previous case reports (10‐21 days), this is likely an idiosyncratic reaction in dogs. One fatal case report, along with the fatal outcome of case 2 in our study, suggests that this can be a life‐threatening drug reaction, although recovery is possible for dogs with a milder clinical presentation. In addition, dog 2 had been administered levetiracetam at the same time as zonisamide, and although there are no reports of a hepatopathy associated with levetiracetam in the veterinary or human medical literature, and acute idiosyncratic reaction cannot be ruled‐out.

One dog had abnormal serum ALT activity 15 days after administering zonisamide with no accompanying clinical signs, and ALT activity normalized after discontinuation of zonisamide, suggesting the possibility of an acute biochemical hepatopathy. This dog highlights the potential value of rechecking serum liver enzyme activity in the first few weeks after initiating oral zonisamide treatment in dogs even if clinical signs are not present.

We also estimated the incidence of clinical or biochemical hepatopathies in pet dogs treated chronically with zonisamide for at least 5 months. Observed increases in serum ALT (5.1% of treated dogs) and ALP (8.6% of treated dogs) activity were generally mild, although not all dogs had repeated biochemical monitoring. Only 1 dog developed mild hypoalbuminemia (0.85% of treated dogs) and no dogs developed hyperbilirubinemia. Importantly, none of the dogs treated chronically with zonisamide developed any clinical signs associated these biochemical changes.

Our study suggests that increases in serum activity of hepatic enzymes (ALP and ALT) can occur during chronically administration of zonisamide, but at an apparently low incidence and without the development of clinical signs, even with continuation of zonisamide. Unfortunately, the majority of dogs with increased liver enzyme activity during chronically administration of zonisamide did not have further diagnostics performed, such as abdominal ultrasound, urinalysis, or bile acids. Therefore, the biochemical changes detected could have been because of another underlying etiology and unrelated to chronically administered zonisamide. Consistent with our findings, increases in serum ALT or ALP activity are found in 2% to 4% of people chronically administered zonisamide, with no clinically relevant hepatotoxicity reported.^12^

There are several limitations to the current study related to the retrospective study design. In both cases of acute, clinical hepatopathy, acute hepatocellular injury with possible necrosis was suspected but was not histopathologically confirmed. Not all treated dogs had biochemical testing performed during the first 3 months of zonisamide administration. In the chronically treated group, biochemical screening was inconsistent, measurement of bile acids was not performed, and some dogs were lost to follow‐up. Additionally, it is possible that some dogs in the chronic administration group had acute, albeit subclinical, biochemical changes that were missed if bloodwork was not performed. For future prospective studies, consistent measurement of liver enzyme activity is needed to clarify the true incidence of biochemical hepatopathy in dogs treated with zonisamide and to define the number needed to test to determine the value of routine biochemical monitoring in these dogs.

In conclusion, our study found an apparent incidence of 0.52% for acute, likely idiosyncratic clinical hepatopathy during administration of zonisamide PO in 384 dogs. Because of the potentially life‐threatening nature of acute hepatopathy, careful clinical monitoring and follow‐up biochemical testing is essential during the first month of treatment with zonisamide. We found no evidence of clinically relevant hepatopathies in dogs treated with zonisamide for 5 months or longer.

## CONFLICT OF INTEREST DECLARATION

Lauren A. Trepanier serves as Associate Editor for the Journal of Veterinary Internal Medicine. She was not involved in review of this manuscript. No other authors have a conflict of interest.

## OFF‐LABEL ANTIMICROBIAL DECLARATION

Authors declare no off‐label use of antimicrobials.

## INSTITUTIONAL ANIMAL CARE AND USE COMMITTEE (IACUC) OR OTHER APPROVAL DECLARATION

Authors declare no IACUC or other approval was needed.

## HUMAN ETHICS APPROVAL DECLARATION

Authors declare human ethics approval was not needed for this study.
